# Music listening in families and peer groups: benefits for young people's social cohesion and emotional well-being across four cultures

**DOI:** 10.3389/fpsyg.2014.00392

**Published:** 2014-05-08

**Authors:** Diana Boer, Amina Abubakar

**Affiliations:** ^1^Department of Social Psychology, Institute of Psychology, Goethe University FrankfurtFrankfurt, Germany; ^2^Department of Cross-Cultural Psychology, Tilburg UniversityTilburg, Netherlands

**Keywords:** music, family rituals, peer groups, culture, social cohesion, emotional well-being

## Abstract

Families are central to the social and emotional development of youth, and most families engage in musical activities together, such as listening to music or talking about their favorite songs. However, empirical evidence of the positive effects of musical family rituals on social cohesion and emotional well-being is scarce. Furthermore, the role of culture in the shaping of musical family rituals and their psychological benefits has been neglected entirely. This paper investigates musical rituals in families and in peer groups (as an important secondary socialization context) in two traditional/collectivistic and two secular/individualistic cultures, and across two developmental stages (adolescence vs. young adulthood). Based on cross-sectional data from 760 young people in Kenya, the Philippines, New Zealand, and Germany, our study revealed that across cultures music listening in families and in peer groups contributes to family and peer cohesion, respectively. Furthermore, the direct contribution of music in peer groups on well-being appears across cultural contexts, whereas musical family rituals affect emotional well-being in more traditional/collectivistic contexts. Developmental analyses show that musical family rituals are consistently and strongly related to family cohesion across developmental stages, whereas musical rituals in peer groups appear more dependent on the developmental stage (in interaction with culture). Contributing to developmental as well as cross-cultural psychology, this research elucidated musical rituals and their positive effects on the emotional and social development of young people across cultures. The implications for future research and family interventions are discussed.

## Introduction

The positive effects of music for human well-being and social bonding have long been acknowledged by scholars, particularly by ethnomusicologists and anthropologists (e.g., Merriam, 1964; Dissanayake, 2006). Evolutionary theorists emphasize that one central function of music is to mobilize social cohesion and to improve mental health and subjective well-being. These notions have an intuitive appeal that applies to musical family rituals as beneficial social activities fostering family cohesion and emotional development in adolescence. However, scholarly attention to music listening as a family ritual is scarce. Even more pressing, the role of culture in the shaping of musical family rituals and their psychological benefits has been neglected entirely (cf. Miranda et al., 2013). In line with the definition of family rituals (cf. Fiese et al., 2002), musical family rituals are hereby defined as *a set of musical behaviors (engagement and listening) that are reported within a family context and hold symbolic meaning for the family members*.

The current study aims to advance our knowledge concerning family rituals by examining whether and how music listening in families contributes to family cohesion and adolescents' emotional well-being, and what role the cultural context plays in musical family rituals. With this research, we elucidate musical activities and their positive effects by investigating two important contextual elements in individuals' development: the family as immediate environment (micro system), and culture as a more remote context (macro-context; cf. ecological system theory of human development; Bronfenbrenner, 1979, 1986). Furthermore, we examine music in families as opposed to in peer groups as another important socialization context. We first elaborate the effects of music listening on emotional well-being, and then explore the functions of musical effects in families and peer groups across cultures and developmental stages of young people.

### Music listening and well-being

One of the most pervasive (and well-studied) functions of music listening exhibits its impact on human emotions and well-being (e.g., Juslin and Västfjãll, 2008; Juslin and Sloboda, 2010). Across cultural contexts music engagement has been associated with psychological well-being across the lifespan (e.g., Ruud, 1997; Morinville et al., 2013). The relationship between music listening and psychological well-being (e.g., depression, life satisfaction and positive affect) has been investigated in two main research streams^1^: (a) self-selected uses of music including emotional regulation, and (b) the physiological and emotional reactions to music exposure.

Among the research investigating music listening and its effects on psychological well-being via self-selected uses of music (including emotion regulation strategies), Laukka (2007) showed that the elderly in Sweden listen to music frequently and exhibit a variety of listening strategies related to emotion regulation. Furthermore, some of the listening strategies were positively associated with psychological well-being. Besides these effects on emotional well-being, further research showed positive effects of music listening on global happiness. Among Canadian adolescents, Morinville et al. (2013) found that self-determined music listening was associated with higher global happiness (assessed as positive and negative affect, and life satisfaction). While these studies focused on positive psychological outcomes, research on psychopathological outcomes suggests that music listening can be utilized as a problem-oriented coping strategy that can lower depression levels in adolescent girls (Miranda and Claes, 2009). From these studies we can conclude that music listening contributes to positive youth development and well-being. Boehnke et al. (2002) argued and found that adolescents actively employ music listening as a means of achieving their developmental aspirations. The striving for physical and psychological well-being and happiness serves as one of the most prevalent drivers of human actions, and music is unique in its pleasure and reward effects.

The physiological, physical, and emotional effects of music have been receiving growing recognition. While listening to pleasant music, specific brain regions are triggered, activating reward, autonomic response, and cognitive processing (Menon and Levitin, 2005). The authors argue that their study offers insights into the mystery of “why listening to music is one of the most rewarding and pleasurable human experiences” (Menon and Levitin, 2005, p. 175). The mechanisms involved in the rewarding and emotional experiences while listening to music have evolved gradually during evolution due to the involvement of distinct brain functions (Juslin and Västfjãll, 2008). These mechanisms involve brainstem reflex, evaluative conditioning, emotional contagion, visual imagery, episodic memory, and musical expectancy (Juslin and Västfjãll, 2008). Given the complex interplay of various brain regions when listening to music, the physiological and physical reactions to musical exposure (Hodges, 2009) are likely to enact their impact not only as positive situational effects, but also on long-term well-being when music is implemented in various life situations and contexts—including the family. The process by which music is associated with emotional well-being seems to be both gradual and cumulative.

In sum, listening to music serves in-depth emotional functions and activates brain regions that provide the rewarding effects of music listening. However, much of the research in this domain has assessed un-contextualized (or laboratory) music listening, whereas most musical engagement and listening routines happen within social contexts and environments. Hence music gives shape and receives importance by the contexts in which the listening behavior is enacted. Most pressing, previous research has not provided empirical evidence for contextualized musical rituals, and the way in which ritualized music listening in different contexts serves the functions of enhancing emotional well-being. Music listening is one of the most frequent and pervasive leisure activities which takes place within nested contexts during adolescent development. Family and peer contexts are the most important and immediate environments that shape individuals' development (Bronfenbrenner, 1979, 1986). The cultural context, on the other hand, presents a more remote but nevertheless influential context factor that is likely to shape micro-context processes (Bronfenbrenner, 1979). In the following sections, we elaborate on the influence of music in family contexts and how musical engagement in families positively influences young people's functioning.

### Music listening in families

Though the emotional aspects of music listening have been emphasized in the literature, music also serves strong social functions (Boer et al., 2011, 2012; Boer and Fischer, 2012). People bond over musical activities and shared music references (Selfhout et al., 2009; Boer et al., 2011), and music contributes to the development of collective identities (Tarrant, 2002; Tarrant et al., 2002; Boer et al., 2013). The need to belong is a universal basic motivation (Maslow, 1970; Ryan and Deci, 2000). When people feel accepted, liked and part of a group, they experience enhanced emotional and psychological well-being and better stress relief (Häusser et al., 2012). The role of music in enhancing social cohesion, as well as individual and collective well-being, has attracted much scholarly attention from musicologists, anthropologists, psychologists and sociologists. Music is one of the most important topics in adolescent life, and music is listened to in many social contexts. Music is therefore very likely to be a major theme in contributing to family cohesion, which in turn will benefit young people's well-being.

The family is the first socializing agent, where most of the early bonding and attachment takes place. Within the family context, music has been found to be the most widely used leisure activity, and it is part and parcel of everyday occurrence. Moreover, music is an important component of most families' routines and rituals. The use of music as part of family rituals starts very early in life, contributing to strong emotional bonds (Parncutt, 2009; Trehub, 2009). Across cultural contexts, the singing of lullabies is part of children's bedtime rituals (Unyk et al., 1992; Gregory, 1997; Trehub, 2009). As children grow older, parents shift from singing *to* them to singing *with* them. This actively involves them in the musical experiences, which adds functional value. For instance, a study of 10 families with 3-year-old children observed that “*families used singing to “make special” routine activities and to create and maintain traditions*” (Custodero, 2006, p. 37). This is later maintained through other activities, with families listening to music during special events, as part of their religious engagement, as part of leisure activities and during get-togethers (Boer et al., 2012). Despite the central role of both the family and music in people's lives, there is surprisingly little research focusing on the function of music within the family unit. The limited research in this area has largely focused on the use of music in the early years of life (e.g., Trehub, 2009).

Media psychology, family ethnographies and research on family rituals have emphasized the role of TV-watching as a family ritual (Lull, 1980; Rubin, 1983; Fiese et al., 2002). During the time the family spends together, TV-watching is often a ritualized aspect of family routines and organization of family activities (Dubas and Gerris, 2002). This form of media consumption serves the functions of facilitating social interaction and bonding, as well as for family entertainment (according to the uses-and-gratification in media psychology; e.g., Rubin, 2008). Hence, media uses are embedded in family routines and rituals, as is music listening (Boer et al., 2012). Some existing evidence indicates that music remains an important part of the family identity, a way of transmitting family values, norms, and culture, and enhancing family cohesion. A recent study highlights the impact that the music children were exposed to during childhood has on their emotional experiences, and the reminiscence bumps aroused by the same music years later (Krumhansl and Zupnick, 2013). The authors suggest that the prevalence of music in the home environment contributes to the shaping and triggering of positive autobiographical memories. Furthermore, this process may feed into intergenerational cultural transmission entailing musical as well as general cultural values. Another study showed that music listening was associated with neurobiological pathways affecting social affiliation and communication (Ukkola-Vuoti et al., 2011). In a family-based association analysis of Finnish families, the arginine vasopressin receptor 1A (AVPR1A) gene haplotypes were positively associated with active current and lifelong listening to music. This study is particularly relevant to the social cohesion function of music, because the AVPR1A gene is associated with social communication and attachment. Results from this study seem to imply that music listening genetically co-occurs with intrinsic attachment behavior and social cohesion.

However, many questions remain unanswered. What is the impact of musical family rituals on family cohesion? Does musically enhanced family cohesion positively influence young people's well-being? We propose that musical family rituals are positively related to family cohesion, based on their ability to enhance social bonding (Boer et al., 2011) and to create social cohesion and identification (Tarrant et al., 2002). Moreover, musical rituals in families are likely to impact on well-being, in particular on emotional well-being due to the primacy of emotional effects (Juslin and Västfjãll, 2008) and the intra-individual functions (Boer and Fischer, 2012; Boer et al., 2012) of music. The investigation into the direct and indirect effects in family rituals has been prompted by family rituals researchers aiming to disentangle underlying mechanisms and advance theory development (Fiese et al., 2002). Crespo et al. (2011) family rituals model seems particularly relevant for the development of our research model. Their multiple mediation model of family rituals proposed and found that family ritual meanings contribute to (parents' and adolescents' perceptions of) family cohesion, which in turn positively impact on adolescents' well-being. Similarly, we propose that musical family rituals positively relate to emotional well-being due to their effects on family cohesion, because individuals who are embedded within well-functioning family contexts are likely to adapt well to developmental demands and develop resilience against emotional drawbacks during development. Alternatively, musical family rituals could also contribute simultaneously and directly to greater cohesion and better emotional well-being, because music enacts a strong direct impact on emotions and well-being. We will assess both possibilities using structural equation modeling.

### Music listening in peer groups

The peer group serves as another important micro-context in which individuals' development is embedded (Bronfenbrenner, 1979; Bronfenbrenner and Morris, 2006). Among adolescents and youth the research focus has largely been on the use of music within the context of peer groups. This line of research has partially been informed by two facts. First, at adolescence there is a developmental shift. Peer involvement becomes increasingly salient, in terms of engagement and shared activities, as one tries to gain autonomy from the family (Steinberg and Silverberg, 1986). Second, from early adolescence throughout young adulthood, a significant amount of time and money is spent on musical activities (Selfhout et al., 2009). Many of these musical activities are shared with members of the peer group. Shared musical preferences and activities contribute to friendship formation in adolescence (Selfhout et al., 2009) and in young adulthood (Boer et al., 2011). Given these two factors, it is understandable that peer-related activities have had more prominence in research than the family. However, developmental research shows that although peer group involvement becomes more intense at adolescence, this relationship does not weaken the parental one. Instead, family rituals receive more symbolic meaning with increasing cognitive development (Fiese et al., 2002). Consequently, it would be more informative to study musical activities within both family and peer group contexts simultaneously, in order to gain better insights into the social functions of music and their influence on well-being.

One of the few studies looking at music in the context of family and peers was conducted by Miranda and Gaudreau (2011). A key research question in this study was the extent to which congruence in musical taste with one's parents and friends enhanced well-being. The study observed that congruence in music taste with both parents and peers was associated with lower levels of negative affect, while congruence with parental taste in music was associated with higher levels of positive affect. These findings highlight the relevance of music in families and peer groups, and its impact on emotional well-being. We predict that musical family rituals and peer rituals are associated with family and peer cohesion respectively, and with emotional well-being (Musical ritual hypothesis 1). Based on the premise that both families and peer groups are relevant socialization agents impacting on the emotional development of young people, we propose that musical rituals in both contexts contribute to emotional well-being via enhanced social cohesion (Musical ritual hypothesis 2).

### Families, culture, and music during development

Music can be found in all cultural contexts across the world. Numerous studies suggest both universal and cultural-specific uses of music across the world (e.g., Merriam, 1964). However, there have been limited studies examining the cross-cultural uses of music and its functions in different contemporary contexts (Boer and Fischer, 2012; Boer et al., 2012, 2013). Boer et al. (2012) study involving six countries from three continents observed cross-cultural differences in the social functions (values, social bonding, dancing) and socio-cultural functions (cultural identity, family bonding, political attitudes) of music. For instance, participants from Kenya and the Philippines seem to have the strongest emphasis on social and socio-cultural functions of music compared to the other cultural samples. Kenyan and Filipino samples experienced family bonding through music most strongly, followed by Mexico and New Zealand, while participants from Germany and Turkey seem to experience musical family bonding less frequently through musical activities. Further analysis indicated that cultural values, such as individualism-collectivism and secularism-traditionalism, explained cross-cultural differences in the uses of music. Listeners from more collectivistic and traditional cultures used music more frequently for expressing values, cultural identity, and family bonding.

During adolescence, individuals mature and develop their personal identity, a process called individuation (Youniss and Smollar, 1985; Olaf and Buhl, 2008). This means that the influence of families—albeit that they remain important throughout one's life-span—decreases to some degree during the transition to adulthood. We therefore expect that musical family rituals will be more strongly associated with younger participants' family cohesion and emotional well-being than for older participants (Developmental hypothesis).

While families are salient across all cultural contexts, there are distinct differences in terms of values and expectations regarding the amount of time and level of engagement in family-related activities by adolescents and young adults. The differences in values and expectations are related to the degree to which a society emphasizes group cohesion, harmony and interdependence (Markus and Kitayama, 1991; Kagitcibasi, 2005). In collectivistic cultures, where there is a relatively high emphasis on families and social bonds, youth and adolescents start the process of individuation later (Dwairy and Achoui, 2006; Dwairy et al., 2006), and tend to spend more of their leisure time within their families compared to those from individualistic cultures, where there is more emphasis on autonomy and personal goals. For youth and adolescents from these individualistic cultures, the process of individuation starts early. Consequently, it would be expected that these youths may spend more time with their peers (Youniss and Smollar, 1985) and engage with them in activities like music listening. Based on this, one would expect that family cohesion contributes more strongly to the emotional well-being of young people in traditional/collectivistic societies compared to young people from secular/individualistic contexts (Culture hypothesis 1), whereas peer cohesion might be more important for emotional well-being in secular/individualistic settings compared to traditional/collectivistic contexts (Culture hypothesis 2).

As previously elaborated, peer groups receive earlier developmental influences in more secular/individualistic cultures compared to more traditional/collectivistic societies. In more traditional societies, peer groups are also important, but their impact on adolescents' emotional development may receive more weight at a later developmental stage. We therefore predict that older participants from collectivistic cultures show a stronger association between musical peer rituals and peer cohesion, as well as emotional well-being, compared to younger participants (Culture-sensitive developmental hypothesis 1), whereas in individualistic cultures, younger participants are expected to show a stronger association between musical peer rituals and peer cohesion as well as emotional well-being compared to older participants (Culture-sensitive developmental hypothesis 2).

### The present study

This paper explores the role of music listening as a family ritual and in peer groups across four cultures located in Africa, Asia, Oceania, and Europe. More precisely, we investigate the benefits of music listening in families for family cohesion and emotional well-being in adolescence. Furthermore, we distinguish the psychological benefits of musical family rituals from music in peer groups. We report on cross-sectional data from Kenya, the Philippines, New Zealand, and Germany. In each of the countries we have studied, there is a strong mix of traditional and modern music being listened to. For instance, in the last 30 years New Zealand has experienced an increased popularity of fusion styles of Western and traditional Maori music. Some of the most successful bands blend different musical genres. In Germany, classical music is enjoyed alongside popular music that has been influenced by American/British music. The same can be said for Kenya and the Philippines, where both traditional and Western styles have merged into modern, culture-specific styles. For instance, in the Philippines there are three streams of music: an indigenous musical tradition, which is influenced by the old Asian cultural elements, the Spanish/European-influenced music, and the American-influenced music largely expressed in popular music entailing culture-specific music genres such as Pinoy Rock (for more details see Boer et al., 2013).

**Figure 1:**
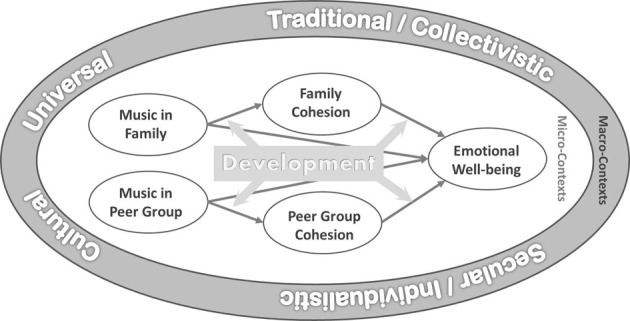
**Culture-sensitive developmental model of music in families and peer groups and their effects on family cohesion, peer group cohesion, and on emotional well-being**.

Kenya and the Philippines are cultural contexts which emphasize traditionalism over secularism (secularism vs. traditionalism scores: Kenya = −0.17, the Philippines = 0.06; World Value Survey, 1981–2008), and collective values over individualistic values (individualism scores: Kenya = 27, the Philippines = 32; Hofstede, 2001). On the other hand, New Zealand and Germany value secularism over traditionalism (secularism vs. traditionalism scores: New Zealand = 1.24, Germany = 0.61; World Value Survey, 1981–2008), and individualism over collectivism (individualism scores: New Zealand = 79, Germany = 67; Hofstede, 2001). We sample participants from these four cultural contexts due to the relevance of their cultural differences on the impact of social cohesion, and the effects of musical rituals on emotional well-being. In our study we anticipate to find cross-culturally similar as well as variable effects. We interpret cross-cultural comparisons according to their implications for levels of universality (Norenzayan and Heine, 2005). In addition to cross-cultural comparisons of the proposed effects, we explore differences between adolescents (below the age of 20 years) vs. young adults (aged 20–29 years). While we expect general developmental patterns with respect to musical family rituals and their associations, we anticipate culture-specific patterns in musical peer group rituals. Our overall research model is depicted in Figure 1. We postulate a hypothetical model which indicates that both family and peer music listening contribute to family and peer cohesion, which in turn is associated with emotional well-being. The relationship between musical ritual, family/peer cohesion and emotional well-being is moderated by the developmental process. Moreover, the relationship between musical rituals and outcomes is shaped by the broader cultural context. The influence of these different cultural contexts is likely to contribute to a pattern where both cross-cultural invariant and culture-specific patterns of relationship between the three variables are elicited. In sum, we propose and test seven hypotheses:

*Musical ritual hypothesis 1*: Musical family and peer rituals will be positively associated with family and peer cohesion respectively, as well as with emotional well-being.
*Musical ritual hypothesis 2*: Musical family and peer rituals will relate to family and peer cohesion respectively, and this in turn will be associated with enhanced emotional well-being.
*Culture hypothesis 1*: In (samples from) collectivistic cultures, family cohesion will be more strongly associated with emotional well-being compared to (samples from) individualistic cultures.
*Culture hypothesis 2*: In (samples from) individualistic cultures, peer cohesion will be more strongly associated with emotional well-being compared to (samples from) collectivistic cultures.
*Developmental hypothesis*: Adolescents will show a stronger association between musical family rituals and family cohesion, as well as emotional well-being, compared to young adults.
*Culture-sensitive developmental hypothesis 1*: In (samples from) collectivistic cultures, young adults will show a stronger association between musical peer rituals and peer cohesion, as well as emotional well-being, compared to adolescents.
*Culture-sensitive developmental hypothesis 2*: In (samples from) individualistic cultures, adolescents will show a stronger association between musical peer rituals and peer cohesion, as well as emotional well-being, compared to young adults.

## Materials and methods

### Data collection and participants

In total, we sampled 760 adolescents and young adults between the ages of 13 and 29 from Kenya, the Philippines, New Zealand and Germany. 436 participants were adolescents below the age of 20, and 324 participants were young adults aged 20–29 (see Table 1 for details). The Kenyan participants were sampled at high schools (boarding schools) and universities located in the Nairobi and Mombasa areas. Filipino students were sampled at a university in Dumaguete. In New Zealand, participants were recruited on the campus of a university in Wellington and online. Similarly, in Germany participants were recruited at universities in Hamburg and Leipzig, as well as via online data collection. The gender distribution of the samples was mostly equivalent; the average age differed across the cultural samples (Table 1). The age difference may have occurred due to different educational systems and entry age at universities. In each sample, more than half of the participants were actively involved in music-making by playing instruments or singing; however, this distribution also differed across the samples. In order to account for those sample differences, we repeated all model tests including age, gender, and musicianship as control variables. In addition, we conducted age-group comparisons for assessing developmental effects (adolescents vs. young adults) in two of the cultural samples (Kenya and Germany), which allow this test due to sample sizes (Table 1).

**Table 1:**
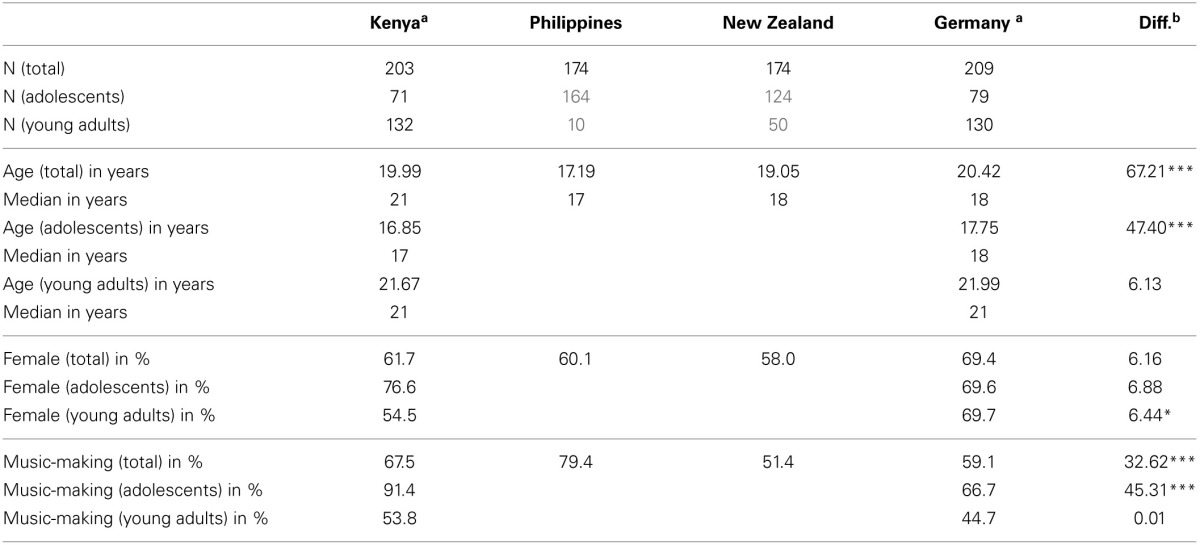
**Sample descriptive**.

^a^Samples are analyzed in total and comparing the two age-groups of under 20 year olds (adolescents) vs. 20–29 year olds (young adults); ^b^age differences are tested using F-statistics, frequency differences are tested using χ*^2^* tests. *p < 0.05, *** *p* < 0.001.

*Gray values refer to sub-sample sizes (in the Philippines and New Zealand) that are too small for age-group analysis*.

This anonymous cross-sectional survey study was approved by the School of Psychology Human Ethics Committee at Victoria University of Wellington, New Zealand [application number: 0800DEC(0000015377)], where the first author conducted her doctoral studies.

### Measures

We assessed musical rituals in families and peer groups, participants' social cohesion as indicators of affiliation with their families and peer groups, and their emotional well-being with regard to positive affective states experienced during the previous few weeks. The mean values are presented for descriptive purposes in Table 2.

**Table 2:** **Mean values, standard deviations (in brackets), and internal consistencies^2^ (Cronbach's alphas in italics) of study variables and correlations (for combined sample)**.

	**Kenya**	**Philippines**	**New Zealand**	**Germany**	1	2	3	4	5
1. Musical family rituals	4.27 (1.71)	4.42 (1.54)	3.63 (1.41)	3.02 (1.45)	1				
	*0.75*	*0.90*	*0.88*	*0.88*					
2. Musical peer rituals	4.75 (1.44)	5.34 (1.25)	4.65 (1.14)	4.56 (1.43)	0.44^***^	1			
	*0.74*	*0.87*	*0.77*	*0.87*					
3. Family cohesion	4.80 (1.76)	5.50 (1.29)	5.50 (1.51)	4.32 (1.54)	0.34^***^	0.09^*^	1		
	*0.45*	*0.75*	*0.87*	*0.78*					
4. Peer group cohesion	3.78 (1.78)	4.18 (1.42)	5.81 (1.07)	5.01 (1.17)	−0.01	0.15^***^	0.25^***^	1	
	*0.67*	*0.74*	*0.72*	*0.71*					
5. Affective well-being (sum score)	32.69 (9.13)	34.76 (6.99)	33.73 (7.16)	34.84 (6.72)	0.17^***^	0.25^***^	0.15^***^	0.12^**^	1
	*0.84*	*0.87*	*0.87*	*0.84*					

* p < 0.05,

** p < 0.01,

*** *p < 0.001*.

#### Musical rituals

The RESPECT–Music scale (Boer et al., 2012) was used to assess musical rituals in families via the family function of music (four items) and in peer groups via the peer function of music (five items). Example items for musical family rituals are “I like talking to my family about music,” “I enjoy listening to music with my family,” and “Music allows me to have a common interest with my family.” Example items for musical peer group rituals are “I meet with friends and listen to good music,” “Going to concerts and listening to records is a way for me and my friends to get together and relate to each other,” and “Listening to music with friends is a way of sharing good old memories of our lives.” Participants indicated the degree to which the item statement applied to their experiences with music from “1—not at all” to “7—to a great extent.” The internal consistencies of the two scales for the whole sample were satisfactory (family: *alpha* = 0.85; peer group; *alpha* = 0.82).

#### Social cohesion

Social cohesion in the family and peer group were measured using two-item indicators based on the relational construal sub-scales from Harb and Smith's (2008) Six-fold Self-Construal Scale. Two items measured family cohesion (vertical-relational construal): “My thoughts and beliefs are most attuned with my family”; “My identity is mostly defined by my belonging to my family.” The same two item phrasings measured peer group cohesion (horizontal-relational construal): “My thoughts and beliefs are most attuned with my friends”; “My identity is mostly defined by my belonging to my friends.” Participants indicated their agreement with the statements on a 7-point Likert scale from “1—strongly disagree” to “7—strongly agree.” The overall internal consistencies of the two scales were satisfying considering the low number of items (family cohesion: *alpha* = 0.71; peer cohesion: *alpha* = 0.77).

#### Emotional well-being

We measured the positive well-being aspects of the Positive and Negative Affect Schedule (PANAS; Watson et al., 1988). Participants indicated the extent to which they experienced 10 positive emotions (e.g., strong, inspired, determined) during the previous few weeks on a 5-point Likert scale ranging from “1—very slightly or not at all” to “5—extremely.” The sum score of positive affect was computed from the 10 items. The internal consistency of the scale for the whole sample was appropriate (*alpha* = 0.85).

### Analytical strategy

First, confirmatory factor analysis (CFA) tests (a) whether the employed measures assess five distinctive concepts, and (b) whether the structural properties and factor loadings are invariant across the four cultural samples^3^. In order to ensure comparability of latent regression weights, measurement invariance is required as equivalence in factor loadings indicates comparable meanings attributed to the latent factors (Van de Vijver and Leung, 1997, 2005). Measurement invariance enables meaningful cross-cultural comparisons of variable associations, while it does not warrant meaningful mean comparisons (Van de Vijver and Leung, 1997, 2005). Therefore, we refrain from interpreting the variable mean scores across the cultural samples (reported for descriptive purposes in Table 2). All analyses were conducted as latent variable structural equation models (SEM) in order to account for measurement errors (in Mplus7).

Second, we test two different SEM models to assess the relationships between music as a family and peer ritual, family and peer cohesion, and emotional well-being: (a) multiple outcome model, and (b) path sequence model. The multiple outcome model tests the predictions of the musical rituals hypothesis 1: the direct effects of musical rituals on cohesion and emotional well-being. This model serves as baseline model for comparison with the path model, which tests the musical rituals hypothesis 2 and evaluates a sequential influence of musical rituals on social cohesion—which in turn contributes to emotional well-being. In order to assess the role of musically facilitated family/peer cohesion in contributing to emotional well-being, we additionally assess their indirect effects via bootstrapped confidence intervals (Preacher et al., 2010). The models were tested for music in both social contexts simultaneously, in order to assess the unique contribution of family and peer rituals while accounting for the respective other effect. We allow for covariation between musical family rituals and musical peer rituals as well as between family cohesion and peer cohesion.

The analyses are conducted on the overall dataset first in order to assess the general tendencies, and we then conduct multi-group analyses in order to test the two models across the four cultural samples assessing the culture hypotheses 1 and 2. Wald χ^2^ tests are conducted to assess cross-cultural equivalence in path coefficients (Muthén and Muthén, 2012). Additionally, we test all models again, entering age, gender, and musicianship as control variables in order to account for sample differences which may confound our findings.

For the assessment of developmental effects proposed in the developmental hypothesis and the two culture-sensitive developmental hypotheses, we run age-group comparisons in two of the four cultural samples (Germany and Kenya). We restrict the age group comparison to these two samples because in the other two cultural samples there would be very small age-group sub-samples (the Philippines, young adults *n* = 10; New Zealand, young adults *n* = 50; see Table 1), which are not sufficiently large for this analysis. In the two samples containing sufficiently large sub-samples, we run multi-group analyses comparing the path coefficients of adolescents (less than 20 years of age) and young adults (between 20 and 29 years of age), using the Wald χ^2^ test.

## Results

### Confirmatory factor analysis

**Figure 2:**
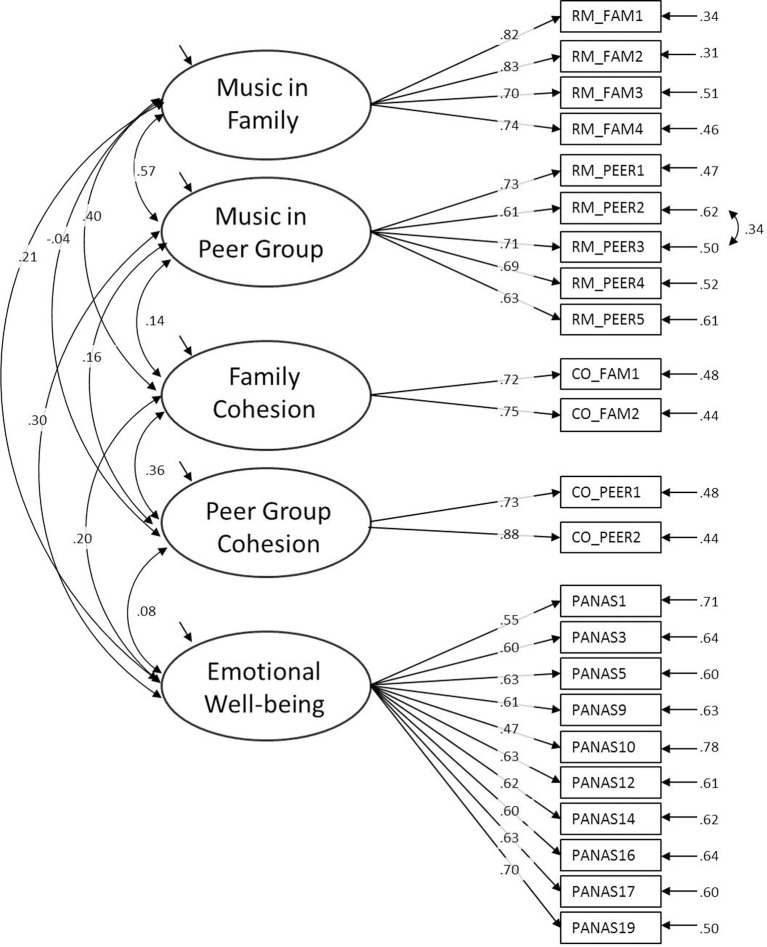
**Confirmatory factor analysis on overall sample data including all five study variables (standardized estimates for factor loadings and error terms)**.

CFA separating the items loading on five latent factors^4^ (all variables involved) revealed satisfactory model fit of the data {χ^2^_(219)_ = 590.92, *p* < 0.001, *CFI* = 0.93, *TLI* = 0.92, *SRMR* = 0.04, *RMSEA* = 0.05 [90% CI = 0.046/0.056]; see Figure 2 for details on factor loadings and error terms}. Multi-group CFA assessed structural and measurement invariance across the four cultural samples. The baseline model (structural equivalence) showed satisfactory fit {χ^2^_(872)_ = 1388.40, *p* < 0.001, *CFI* = 0.91, *TLI* = 0.90, *SRMR* = 0.06, *RMSEA* = 0.06 [90% CI = 0.054/0.066]}, suggesting that the five latent variables are measured in a similar structure^5^ in the four samples. Next, we assessed measurement invariance by constraining the factor loadings to be equal across samples. The model fit of the constrained model indicated invariance of all but one factor loading (Δχ^2^/Δ*df* = 2.23, n.s.; Δ*CFI* = −0.01). In the constrained model we relaxed one item constraint (first item of musical family rituals: “I like talking to my family about music”) to be freely estimated in the four samples, and we added one co-variation in the New Zealand data of the social cohesion items (the first item of family cohesion co-varied with the first item of peer cohesion) and one co-variation in the German data of the social cohesion items (the second item of family cohesion co-varied with the second item of peer cohesion). Both co-variations are likely to be due to same wording of the items. In sum, the latent measurement of the involved variables and indicators seems appropriately comparable across the four samples (partial measurement invariance; Byrne and Stewart, 2006) so that further analysis based on latent variable modeling is warranted.

### Multiple outcome model

In the overall data, music in families had a medium effect on family cohesion, while its effect on emotional well-being was close to zero (for path coefficients see Table 3). Music in peer groups, on the other hand, showed small effects on peer cohesion and emotional well-being. Comparing the multiple outcome models across the four cultural samples revealed that musical rituals in families and peer groups related to family and peer cohesion, respectively in all four samples, with small to medium effect sizes. This part of the musical rituals hypothesis 1 is therefore supported. The link to emotional well-being, however, showed culture-specific patterns: in the Philippines, only music in families contributed to emotional well-being, whereas in New Zealand, Germany, and Kenya (marginally), only music in peer groups was associated with higher emotional well-being. Despite these differences, the Wald χ^2^ tests indicated equivalence in regression weights (see right column in Table 3). As emotional well-being is related to musical family rituals in only one sample, this part of the musical rituals hypothesis 1 is only partially supported.

**Table 3:** **Results of musical rituals models [standardized regression weights β incl. 95% confidence intervals (95% CI); Wald χ^2^ test (*df* = 3) assessing cross-cultural difference in regression weights; Indirect effects: unstandardized point estimate incl. 95% confidence interval based on 1000 bootstrap iterations, 95% bCI]**.

**Tested model**	**Path**	**Statistics**	**Overall**	**Philippines**	**New Zealand**	**Kenya**	**Germany**	**Diff**.
**MULTIPLE OUTCOME MODEL**								
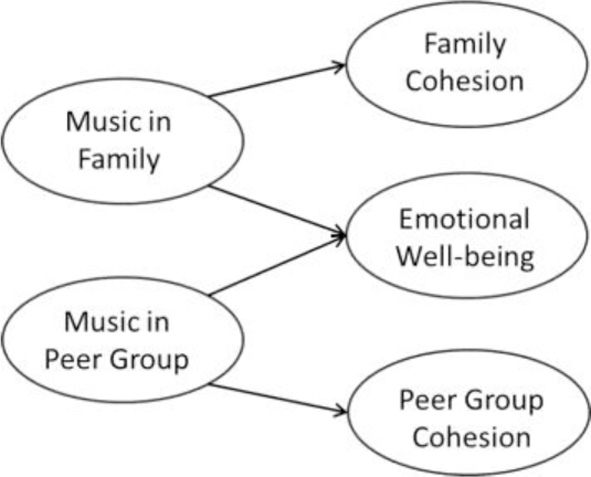
	FAM 1	β	0.43^***^	0.24^**^	0.38^***^	0.37^**^	0.40^***^	2.47
		95% CI	0.34/0.51	0.08/0.40	0.24/0.51	0.1/0.64	0.24/0.57	
	FAM2	β	0.06	0.27^*^	−0.03	0.13	−0.04	4.81
		95% CI	−0.05/0.18	0.04/0.50	−0.25/0.19	−0.17/0.44	−0.22/0.15	
	PEER 1	β	0.28^***^	0.03	0.40^***^	0.28^†^	0.31^**^	5.47
		95% CI	0.16/0.39	−0.22/0.27	0.17/0.63	−0.03/0.58	0.12/0.49	
	PEER 2	β	0.17^***^	0.23^***^	0.35^***^	0.39^***^	0.35^***^	2.19
		95% CI	0.08/0.26	0.08/0.38	0.18/0.52	0.2/0.58	0.18/0.51	
**PATH SEQUENCE MODEL**								
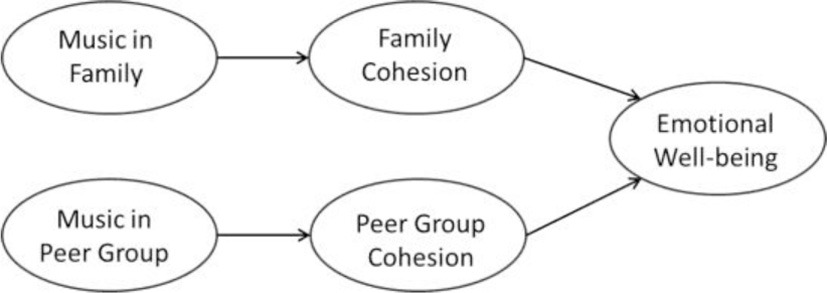
	FAM 1	β	0.42^***^	0.25^**^	0.38^**^	0.55^**^	0.41^**^	1.72
		95% CI	0.33/0.5	0.09/0.41	0.24/0.51	0.33/0.78	0.25/0.57	
	FAM 2	β	0.20^***^	0.16^†^	−0.13	0.62^***^	−0.09	9.44^*^
		95% CI	0.1/0.3	−0.02/0.35	−0.55/0.3	0.31/0.93	−0.32/0.14	
	PEER 1	β	0.15^**^	0.23^**^	0.41^***^	0.37^**^	0.38^***^	5.43
		95% CI	0.05/0.25	0.08/0.38	0.23/0.58	0.18/0.57	0.21/0.54	
	PEER 2	β	0.06	0.00	0.43^†^	−0.19	0.25^†^	6.54^†^
		95% CI	−0.04/0.16	−0.18/0.17	−0.02/0.88	−0.50/0.13	−0.02/0.52	
**INDIRECT EFFECTS MODEL**								
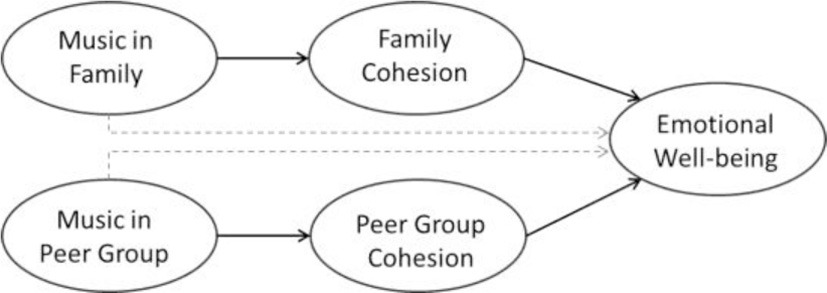
	DIRECT	β	0.01	0.25^*^	−0.07	−0.02	−0.07	4.41
		95% CI	−0.12/0.14	0.02/0.49	−0.36/0.22	−0.39/0.34	−0.29/0.16	
	INDIRECT	point est.	0.020^*^	0.018	0.044	0.185^*^	0.034	–
		95% bCI	0.004/0.040	−0.053/0.095	−0.157/0.282	0.003/6.614	−0.152/0.227	
	DIRECT	β	0.27^***^	0.03	0.38^*^	0.38^*^	0.29^*^	4.42
		95% CI	0.15/0.39	−0.22/0.28	0.08/0.68	0.04/0.71	0.07/0.51	
	INDIRECT	point est.	0.002	−0.078	0.024	−0.119	0.020	–
		95% bCI	−0.006/0.012	−0.007/0.043	−0.203/0.302	−5.269/0.017	−0.103/0.236	

† p < 0.10,

* p < 0.05,

** p < 0.01,

*** *p < 0.001*.

### Path sequence model and indirect effects

Music in families and peer groups contributed significantly to family and peer cohesion, respectively. These effects were present in the overall dataset and across the four cultural samples with equivalent regression weights (see Wald χ^2^ test, Table 3). Family and peer cohesion in turn showed culture-specific associations to emotional well-being (indicated by at least marginally significant Wald χ^2^ tests, see Table 3). Family cohesion was positively related to emotional well-being in Kenya (large effect) and in the Philippines (marginal small effect), whereas this link was not found in New Zealand and Germany, lending support to our culture hypothesis 1. In the latter two contexts there was a marginal positive association between peer cohesion and emotional well-being, in line with our culture hypothesis 2. This link that was not found in Kenya, in the Philippines and in the overall dataset. The path sequence model suggests that musical rituals contribute to both family and peer cohesions across the cultural contexts, whereas family cohesion facilitates emotional well-being in more traditional/collectivistic contexts Kenya and (to a lesser extent) in the Philippines, while peer cohesion seems to foster emotional well-being in secular/individualistic contexts New Zealand and Germany. These results indicate rather culture-specific patterns regarding our musical rituals hypothesis 2, which is specifically tested in the indirect effects analysis.

**Figure 3:**
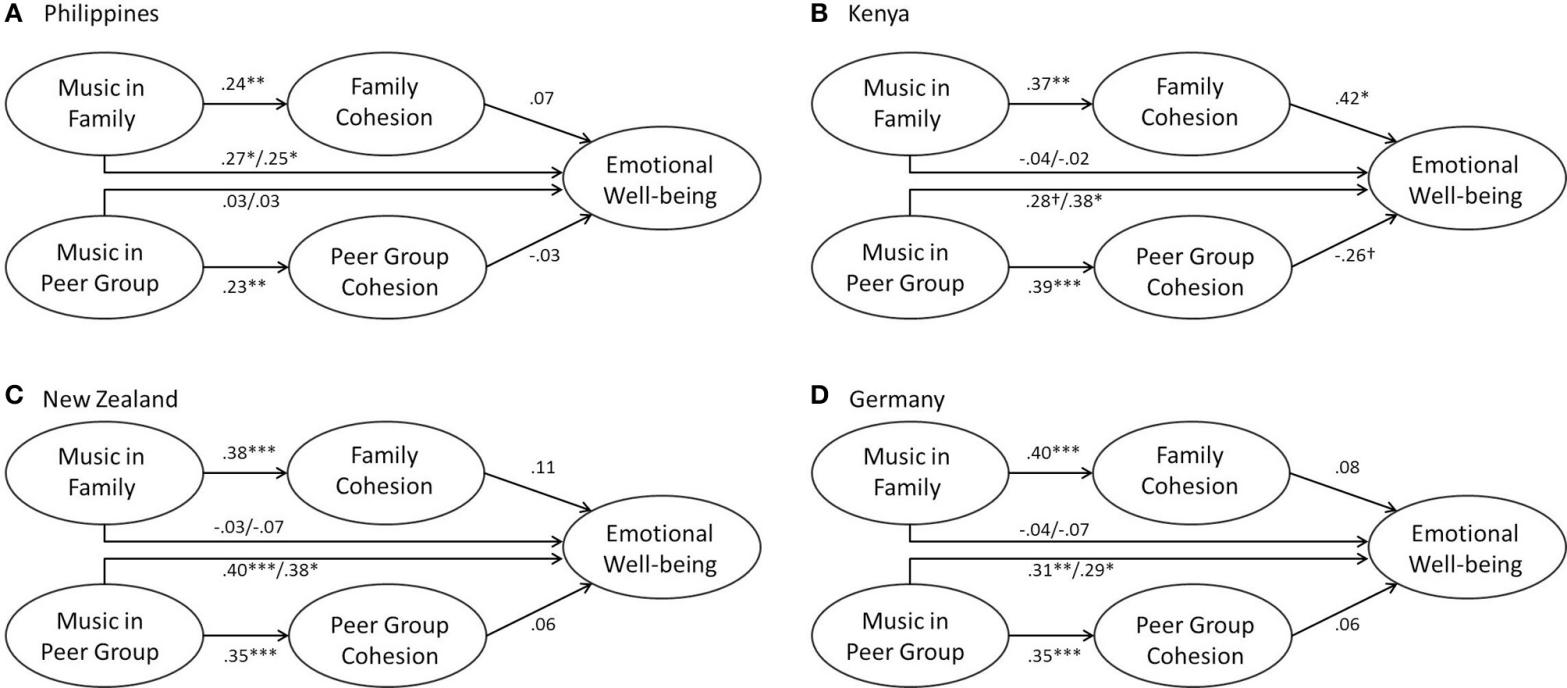
**Indirect effects models including total and direct effects in the four cultural samples**. ^†^*p* < 0.10, ^*^p < 0.05, ^**^p < 0.01, ^***^p < 0.001.

The overall data showed an indirect effect of musical family rituals on emotional well-being via family cohesion, in partial support of musical rituals hypothesis 2. However, when looking at the cross-cultural analyses, this effect occurred only in the Kenyan sample (for details on the path coefficients see Table 3 and Figure 3). The direct effect was significant only in the Filipino sample (Table 3). For musical peer rituals, no indirect effect was found, hence musical rituals hypothesis 2 was not supported with regard to music in peer groups. In contrast, musical peer rituals showed a direct effect on well-being across three cultural contexts (with the exception of the Filipino sample where this effect was not found). In sum, when accounting for musical peer rituals and peer relations, the culture-specific contribution of musical family rituals (as direct or indirect effect) on well-being in more traditional/collectivistic contexts is highlighted. The direct contribution of music in peer groups on well-being appears invariant in two secular/individualistic and one traditional/collectivistic cultural context.

### Model evaluation

We tested the associations of musical rituals on social cohesion and emotional well-being in two different models. The two models (see Table 4) showed satisfying fit with regard to all model evaluation indicators. Multiple outcome model had similar fit (changes did not exceed the benchmarks of Δ*CFI* = −0.01; cf. Vandenberg and Lance, 2000) or slightly better fit (based on significant χ^2^ differences) compared to the paths sequence model. The paths sequence and indirect effect analyses were, however, more helpful in untangling systematic cross-cultural similarities and differences. We therefore conclude that our two-model analysis strategy and the assessment of indirect effects appropriately assessed our research questions.

**Table 4:** **Model fit**.

	**χ^2^**	**df**	**CFI**	**TLI**	**RMSEA (CI)**	**SRMR**
**MULTIPLE OUTCOME MODEL/INDIRECT EFFECTS MODEL**						
Overall sample	608.61^***^	221	0.92	0.91	0.052	0.047
					(0.047/0.056)	
Multi-group analysis	1413.69^***^	884	0.91	0.90	0.060	0.070
					(0.054/0.066)	
**PATH SEQUENCE MODEL**						
Overall sample	701.54^***^	224	0.91	0.89	0.057	0.068
					(0.052/0.062)	
Multi-group analysis	1449.77^***^	892	0.91	0.89	0.062	0.083
					(0.056/0.067)	

CI—90% Confidence Interval of Root Mean Square Error of Approximation (RMSEA);

*** *p < 0.001*.

### Additional analysis

We repeated all analyses adding the control variables age, gender and musicianship as predictors of the outcomes, because these variables may impact on the outcome variables emotional well-being and family/peer cohesion. These additional analyses revealed very similar effects sizes: the correlation between controlled and uncontrolled regression weights were 0.98 for the overall analysis. This vector correlation indicates that the results are stable, and are unlikely to be influenced by the demographic sample variations in gender, age, and musicianship. Furthermore, the regression weights of the control variables gender and musicianship were non-significant in most analyses, indicating that there are no systematic effects that may be missing in our model tests. Age, on the other hand, showed some significant results, which underlines the necessity for the age-group comparisons reported next.

### Age-group comparison

In order to explore developmental patterns, we compared musical rituals and their associations with cohesion and emotional well-being between adolescents and young adults in the Kenyan and German samples. For musical family bonding we did not find support for our developmental hypothesis. In Kenya and Germany, younger and older participants benefited similarly from associations between musical family rituals and family cohesion [e.g., path sequence model: Kenyan adolescents β = 0.51, young adults β = 0.36, χ^2^_(1)_ = 0.38, *p* > 0.10; German adolescents β = 0.43, young adults β = 0.42, χ^2^_(1)_ = 0.21, *p* > 0.10]; the association between family cohesion and emotional well-being was also equivalent across the two developmental stages [Kenyan adolescents β = 0.50, young adults β = 0.20, χ^2^_(1)_ = 1.10, *p* > 0.10; German adolescents β = 0.13, young adults β = 0.01, χ^2^_(1)_ = 0.21, *p* > 0.10].

Developmental differences were found for musical peer bonding. Age-group comparison in the path sequence models revealed that for older participants in Kenya, musical peer rituals were more strongly associated with peer cohesion than for younger participants [multiple outcomes model: 0.51 vs. 0.10, χ^2^_(1)_ = 4.29, *p* < 0.05], whereas the reverse was the case in the German sample [adolescents β = 0.54, young adults β = 0.28, χ^2^_(1)_ = 3.05, *p* < 0.10]. These results are in line with our culture-sensitive developmental hypotheses 1 and 2.

## Discussion

The current study explored whether and how music listening in families and peer groups contributes to young people's family and peer cohesion, and emotional well-being. Furthermore, we examined the role played by the cultural context, and whether musical rituals affect adolescents and young adults differently. Our study revealed that across four cultures music listening in families and peer groups contributes to family and peer cohesion, respectively. The direct contribution of music in peer groups on well-being appears to be applicable in two secular/individualistic and one traditional/collectivistic cultural context. Contrary to these culturally invariant findings, our study revealed that musical family rituals affect emotional well-being particularly in more traditional/collectivistic contexts. Our study contributes the first (to our knowledge) empirical account of music listening in families and peer groups, and its effects on social cohesion and emotional well-being. Contributing to developmental and cross-cultural psychology of family rituals and music, this research elucidated musical rituals and their positive effects in two important socialization contexts (micro systems family and peer group) across four cultures, characterized by tradition/collectivistic vs. secular/individualistic cultural values as more remote context variables (macro-context; Bronfenbrenner, 1979, 1986).

### Music rituals and psychological well-being

Listening to music with family and friends is associated with positive emotions. In line with a vast amount of research on the emotional effects of music-listening (for an overview see Juslin and Sloboda, 2010), our findings suggest that music listening as a social activity relates to more positive emotions being experienced in everyday life. Hence musical rituals contribute to positive emotional well-being or vice versa: individuals who are more positive in their emotional well-being tend to engage in more music listening with their peers and family members. The direct effects of musical rituals in peer groups on emotional well-being received more support from our findings than the indirect effects via social cohesion. Regarding the question of whether music rituals affect emotional well-being directly or via mediational processes of social cohesion, our findings are more in favor of the direct effects. The direct effect of musical peer rituals on emotional well-being seems applicable across cultures: this effect occurred in multiple outcome and indirect effect models across three cultures without differences in effect sizes which suggests similar accessibility of musical peer rituals for experiencing positive emotions (Norenzayan and Heine, 2005). In contrast, the direct and indirect effects of musical family rituals seem culture-specific to the traditional/collectivistic samples from the Philippines and Kenya, respectively.

Music listening as a social activity is associated with strong affiliation and connection. The joint activity in peer groups and families was hypothesized and found to go hand-in-hand with young people's sense of affiliation with their peers and families. While experimental research showed that music listening preferences can create social attraction and bonds among strangers (e.g., Boer et al., 2011), our study provides contextualized evidence for a positive relationship between musical activities and social affiliations in primary socialization contexts. Our cross-sectional study cannot contribute to causal claims—be it an indicator of or a contributor to good relationships—but we pervasively show how important music can be for positive social associations. The effects in the multiple outcome and path sequence models were of similar strength across four cultural contexts indicating evidence for accessibility universal (Norenzayan and Heine, 2005): musical rituals contribute significantly and to a similar extent to social cohesion across the studies cultures. In line with ethno-musicological evidence (Merriam, 1964) our findings are not surprising, considering that music listening is among the most popular leisure activity and conversation topic of adolescents and young adults (Rentfrow and Gosling, 2006; Selfhout et al., 2009). If this important activity is accepted, supported and even related to by peers and family members, music serves as an important vehicle for communication and relationship maintenance.

### Musical rituals and their effects across cultures

The effects of musical family rituals on emotional well-being (when also accounting for peer effects) occurred only in the two more traditional/collectivistic contexts: in the Filipino sample, music in families related directly to more positive emotional experiences, while in Kenya music in families contributed to family cohesion which in turn supports more positive emotional well-being. Music in families is not only particularly prevalent and important in the two traditional/collectivistic contexts (see Table 2), it is also directly or indirectly linked with better emotional well-being. In these cultural contexts, young people who live in families engaging in musical rituals have a better emotional well-being. The reverse may also be the case: the lack of musical rituals in families co-occurs with less positive emotional experiences in young people's everyday lives. These findings are consistent with the traditional family values and collectivistic interdependent self-construals being important facilitators of well-functioning and well-being in these two cultures (cf. World Value Survey, 1981–2008; Markus and Kitayama, 1991; Hofstede, 2001). Although the same items were used in our study for the measurement of musical rituals in four cultures, it is unclear whether the same musical behaviors in families unfold stronger effects on well-being due to more important traditional family values or whether other and closer musical behaviors are enacted in families in these contexts. More in-depths cultural and cross-cultural research is necessary to resolve these questions.

### Musical rituals among adolescents and young adults

Developmental aspects of musical rituals have also been explored across two cultural samples (from Kenya and Germany) by comparing the data of adolescents and young adults. Our developmental hypothesis posited that adolescents compared to young adults show a stronger association between musical family rituals and outcomes, due to the universal developmental trajectory of individuation and increasing independence from the family. However, our data on this did not support the hypothesis. In both samples we did not find a difference between adolescents and young adults. The explanations for these unexpected findings may, however, differ across the two cultural contexts. For the Kenyan findings, the first explanation relates to the opportunity for musical engagement with families, and the second one is related to the process of individuation and separation from family. Firstly, most of the adolescents we sampled were attending boarding schools. Within the boarding school context in Kenya, adolescents are more controlled in their use of and access to music. Living away from their families, they have less opportunity to listen to music together with their parents, siblings, and other family members. At university level there is more opportunity for music listening and participation. We did not collect data on this, but it would be worth testing this potential explanation further. Future research should explicitly ask how much time participants spent listening to music with their families. A second potential issue may arise from the individuation process among emerging adults in Kenya. As noted by theorists such as Kagitcibasi (2005) or Markus and Kitayama (1991), one may expect that among individuals from cultures where interdependence is greatly valued, even in adulthood the strong family ties are kept intact.

Two other possible explanations may account for the unexpected findings in Germany. First, the German context may allow young people a prolonged period of role and identity exploration, which is a phase called emerging adulthood (Arnett, 2000). In this phase, young adults in industrialized contexts are showing a delay in taking up adult roles—partly due to the extended period of tertiary education in the current sample, and the prolonged financial dependence that co-occurs with it. This implies enhanced family dependence and intense interaction with the family members. This explanation relates to the family change model (Kagitcibasi, 2005; Kagitcibasi et al., 2010) which posits that while people may seek autonomy in certain aspects of life, they maintain a strong psychological bond with their families. So one could say that music may be an area in which emerging adults in Germany still actively involve their families—even more so than adolescents who actively try to find psychological independence from their families. A second more speculative explanation is the long term aspects of rituals. If musical activities had already been established as a family ritual in younger years, their effects may emerge more clearly once the young adults have moved out. This means that music as a means of family communication and relationship maintenance may have a longitudinal effect in German families.

Our culture-sensitive developmental hypotheses 1 and 2 received support. Kenyan young adults showed a stronger association between musical peer rituals and peer cohesion, as well as emotional well-being, compared to adolescents. This result is likely to be based on the possibility that individuals in more traditional/collectivistic societies engage in a later individuation, such that peer influences become more important later in their development (young adulthood). For the secular/individualistic setting in Germany, we expected the reverse patterns (earlier individuation leading to stronger peer influence in adolescence vs. young adulthood). Our findings showed that German adolescents' peer cohesion was more strongly affected by musical peer rituals than that of young adults. Hence adolescents in this secular/individualistic culture seem to strive for an early individuation by engaging in close peer contact and using music to maintain and bolster a cohesive and supportive peer affiliation. In later years, the impact of music on peer cohesion seems weakened—different topics may become more important during this developmental stage. In sum, musical family rituals show a consistently strong impact on family cohesion across developmental stages, and musical rituals in peer groups appear more dependent on the developmental stage.

### Limitations

Our study reports on a cross-cultural study and includes data of young people from four national cultures and two developmental stages. The cross-sectional, unrepresentative nature of our samples posits limitations with regard to causal inferences that cannot be drawn, and the limited generalizability of our results. Alternative causal models could also be developed and tested—for instance, the possibility that emotionally well-adjusted young people are more likely to be part of a cohesive peer group which then engages in musical activities together. We based our hypotheses on previous theorizing and empirical evidence of the development of well-being via social cohesion (e.g., Crespo et al., 2011), and the support of musical activities on family and peer group functioning (e.g., Miranda and Gaudreau, 2011). Only comparing late adolescents with young adults provides limited scope for detecting developmental trajectories. Young people in these age-stages are known to be most committed to music listening, and their commitment is arguably comparable across those two stages. This equivalence in musical commitment was an important prerequisite for the age-related comparisons of the musical rituals effects. The findings that this limited dataset revealed are promising, and encourage further empirical testing. Despite its limited generalizability, our study offers initial empirical evidence for an under-researched—yet important—field that holds much potential for future research and therapeutic application.

We conducted comparisons of path coefficients across four cultural groups and across two developmental groups utilizing Wald χ^2^ tests. This test assesses the equivalence of regression weights and, if significant, it indicates that there is a statistically meaningful difference in the path coefficients between the tested groups. Testing equivalence in regression weights is more powerful than simply evaluating whether or not a path coefficient is significant in the different groups, as two significant regression weights could still be of significantly different magnitude. Cross-cultural research methodology has advanced various techniques for assessing equivalence in regression weights (e.g., Van de Vijver and Leung, 2005; Bond and van de Vijver, 2011). However, when the anticipated effect sizes are of small magnitude, Wald χ^2^ test may not be powerful enough for revealing cross-cultural differences, because systematic cross-cultural differences of hypothesized small effects are consequently also small. For such small yet meaningful and systematic cross-cultural variations, multi-level analyses using cultural level predictors of regression weight variation (linkage effects, cf. Bond and van de Vijver, 2011) might be more adequate and powerful in revealing and explaining cultural variations. Nevertheless, assessing the similarity of regression weights also provides insightful hints for interpreting the results according to levels of universality (Norenzayan and Heine, 2005), while these hints require substantive further cultural studies and different methodologies before evidence of universality can be concluded.

### Practical implications

Our work has several theoretical and practical implications. First, given the salience of parenting behavior in shaping adolescent outcomes, there has been a proliferation of research on how parenting influences outcomes. However, most of this research has focused on parenting styles, parental involvement, and monitoring. Little—if anything—has been done on familial musical rituals. Our results indicate the need to expand the theoretical framework guiding our work, so as to include the study of musical rituals in the family and how they relate to other aspects of parenting behavior. Second, musical family rituals present a potentially important bonding process for families. Given the difficulties parents sometimes experience with their adolescents and young adults as they go through the developmental process of individuation, the practice of family rituals, especially musical rituals, may provide a fun and easy approach to maintaining the family bonds and cohesion for adolescents and emerging adults.

Lastly, the therapeutic use of music is well documented, and the potential role of family ritual in intervention work has been implied in previous work. Our study builds further on the existing knowledge base by clearly illustrating the practical value of musical rituals within both family and peer contexts, and elucidates important pathways by which this process benefits adolescents' and young adults' well-being. We show that musical ritual across diverse cultural contexts significantly contributes to the development of a sense of belonging and relatedness, which form a basic motivational need whose fulfillment contributes to psychosocial adjustment (Baumeister and Lear, 1995). Among adolescents and young adults, a key challenge is achieving autonomy while maintaining a healthy sense of belonging to the people and institutions within the salient ecological contexts they operate daily. Numerous studies show that a lack of attachment and connectedness to family, schools, and peers can lead to significant adjustment problems among adolescents (Markham et al., 2003; Shochet et al., 2006; Bond et al., 2007). Our findings indicate that musical rituals can be incorporated into family therapy and school intervention programs aimed at enhancing students' connectedness to these ecological contexts. Moreover, the potentially positive role of musical rituals within intervention programs seems to be generalizable across many cultural contexts. Given the fact that it is a relatively cheap and easy intervention to implement, further work needs to be done to develop and evaluate intervention programs that use musical rituals to enhance cohesion and well-being.

In conclusion, our research answers recent calls for cultural developmental research on music and its effects (Miranda et al., 2013). Contributing to developmental as well as cross-cultural psychology, this research elucidated musical rituals and their positive effects in two important socialization contexts, in four cultures as macro-contexts, and across two developmental stages. Family and peer relations as well as positive emotional experiences in everyday life are strengthened when music is around: listening to music and even talking about one's favorite songs with family and friends is enjoyable and supportive of development. Young people's social and emotional well-being benefits from ritualized musical activities in families and peer groups across different cultures.

### Conflict of interest statement

The authors declare that the research was conducted in the absence of any commercial or financial relationships that could be construed as a potential conflict of interest.

## Footnotes

^1^Music therapy is an additional field of research receiving increasing attention. We, however, do not include music therapy in this review as it has an intervention focus, while this paper intends to focus on the naturally occurring well-being effects of music listening in adolescents' lives. In the closing section, we will discuss the implications of our findings for family therapy.

^2^All Cronbach's alphas are satisfying considering the low number of items, except for the alpha for family cohesion in the Kenyan sample, which seems below the acceptable level of internal consistency. However, the inter-item correlation between the two family cohesion items (*r* = 0.29) exceeded the suggested minimum of 0.20 (Briggs and Cheek, 1986). Hence, the internal consistency of family cohesion in the Kenyan sample can be considered as still acceptable. Furthermore, our main analyses are based on latent-variable modeling taking measurement error into account.

^3^We assessed the goodness-of-fit (in CFA and SEM) according to indicators suggested by Vandenberg and Lance (2000): Chi-square to degrees of freedom ratio (below 3), root mean square error of approximation (RMSEA; equal or below 0.08; standard root mean-square residual (SRMR; equal or below 0.08), Tucker Lewis Index (TLI; above 0.90), and comparative fit index (CFI; above 0.90). Alternative models and invariance levels were evaluated based on model fit (using the same indicators as described above) and the change of fit between nested models using the practical approach of difference between CFI scores. The CFI value should improve above 0.01, if two alternative models are being tested, and the CFI value should not decrease by more than 0.01, if invariance levels are being tested. The classical indicator ΔChi-square is also routinely reported.

^4^The residual terms of two items of the musical peer rituals scale co-varied (standardized estimate = 0.34, *p* < 0.001).

^5^In order to account for culture/language specifics in the factor structures, one culture-specific co-variation between well-being item residual terms was added to the multi-group model in addition to the culture-general co-variation of two musical peer rituals items mentioned in the previous analysis.
